# Quality of Life and its Related Factors Among Iranian Cervical Cancer Survivors

**DOI:** 10.5812/ircmj.4410

**Published:** 2013-04-05

**Authors:** Shahnaz Torkzahrani, Leila Rastegari, Nahid Khodakarami, Alireza Akbarzadeh-Baghian, Kamyab Alizadeh

**Affiliations:** 1 Department of Midwifery, Faculty of Nursing and Midwifery, Shahid Beheshti University of Medical Sciences, Tehran, IR Iran; 2 Department of Midwifery, Faculty of Nursing and Midwifery, Zanjan University of Medical Sciences, Zanjan, IR Iran; 3 Department of Biostatistics, School of Medicine, Shahid Beheshti University of Medical Sciences, Tehran, IR Iran; 4 Department of Obstetrics and Gynecology, Gynecologic Cancer Research Center, Imam Hossein Hospital, Shahid Beheshti University of Medical Sciences, Tehran, IR Iran

**Keywords:** Uterine Cervical Neoplasms, Quality of Life, Questionnaires

## Abstract

**Background:**

Cervical cancer is the main cause of malignancy-related death among women living in developing countries

**Objective:**

The aim of this study is to evaluate the quality of life (QOL) among Iranian cervical cancer survivors and its relationships with demographic and disease related factors.

**Patients and Methods:**

A descriptive correlational study was carried out on 65 consecutive cervical cancer survivors in three different oncology centers related to Shahid Beheshti University of Medical Sciences, Tehran. The QOL was evaluated using three different standard questionnaires: 1) EORTC QLQ-C30 for patients with malignant tumors; 2) EORTC QLQ-CX24 for cervical cancer patients; and 3) SSQ for assessing the social support. The data was obtained by telephone interviews. The test-retest reliability and internal consistency of the scales were examined. Cronbach's alpha was calculated to assess internal consistency among items. Content validity was assessed to review the scales.

**Results:**

Cervical cancer survivors stated a good QOL. However, its score was negatively associated with symptoms including short breathing, lack of appetite, nausea and vomiting, sleep disorders, peripheral neuropathy, and menopausal symptoms. Also, there was a positive association between QOL and economic conditions as well as QOL and social functioning.

**Conclusions:**

Although, the QOL in cervical cancer survivors was good, treatment of related symptoms can influence the QOL and improve the care of these patients.

## 1. Background

Cervical cancer is the main cause of malignancy-related death among women living in developing countries. It has been estimated that annually there are 500,000 women with cervical malignancies and 270,000 deaths related to cervical cancer worldwide. The developing world bears the burden of about 80% of the disease and 88% of its related mortalities, where 13% of malignancies in women are cancer of cervix (1, 2). Although, the diagnosis and treatment of cervical cancer has been developed recently, there are important consequences from the disease and its treatment among survivors, especially the impact on quality of life (QOL). The chronic nature of the disease can affect the QOL of these patients and their families. Also, functional disorders may be resulted from therapies such as surgery, which could involve the female genital anatomy; radiotherapy which could damage the vaginal mucosa and epithelium; and chemotherapy which could induce some side effects like nausea, vomiting, diarrhea, constipation, microsites, weight changes and hormonal changes. In addition, psychological factors are usually involved in these patients including incorrect beliefs about the origin of cancer, changes in self-image, low self-esteem, marital tensions, fears and worries which all can affect the patients’ QOL (3). Nowadays, the interests in investigation of the QOL in different diseases are increasing due to its potential value in identifying patients’ problems and discovering the challenges and planning for the health systems. Considering the impact of cancer on QOL and also long-time survival of cervical cancer patients due to screening methods and its early treatment, study on the field of QOL and its related factors are important. This is the first study in Iran to evaluate the QOL and its associated factors among cervical cancer survivors.

## 2. Objective

The aim of this study is to evaluate the quality of life (QOL) among Iranian cervical cancer survivors and its relationships with demographic and disease related factors.

## 3. Patients and Methods

This descriptive correlational study included all consecutive patients with primary cervical cancer who had been treated in three oncology centers in Tehran, Iran (Taleghani Hospital, Imam-Hossein Hospital, and Shohadaye-Tajrish Hospital) and had no other diagnosed malignancies as well as no psychological disorders. Also, their treatment courses were completed at least 8 months before the study, but no more than 7 years (between 2001 and 2009). Data were collected during Aug and Sep 2009, through phone interviews, at a single time. Three questionnaires were used: Initially, an instrument for socio-demographic and clinical characteristics was applied. For the evaluation of QOL, the translated version of European Organization for Research and Treatment of Cancer (EORTC) Core 30 Quality of Life Questionnaire (QLQ-C30), version 3.0 (a QOL instrument for all cancers which was previously validated and found to be reliable in Iran) (4) and EORTC QLQ-CX24 (a cervical cancer-specific QOL module which was translated, culturally adapted, and validated in our study) (5) as well as a 10-item social support questionnaire (SSQ) (6) were used. The EORTC QLQ-C30 consists of 30 questions (4-Likert scale) which cover physical functioning, role functioning, emotional functioning, cognitive functioning, social functioning, global QOL, energy/fatigue, nausea and vomiting, pain, and six single items (short of breath, sleep disturbance, lack of appetite, constipation, diarrhea, and financial problems). The EORTC QLQ-CX 24 consists of 24 questions (4-Likert scale) including three multi-item scales on symptom experience, body image, and sexual/vaginal functioning and six single-item scales covering statements on lymphedema, peripheral neuropathy, menopausal symptoms, sexual worry, sexual activity, and sexual enjoyment. The study protocol was approved by the ethics committee and the institutional review board of the Shahid Beheshti University of Medical Sciences, Tehran, Iran. An informed consent was obtained from participants before entering into the study. Results are given as mean and standard deviation (SD) for continuous variables and number (percent) for categorical variables. The total score for each segment is given as percentage and was calculated by dividing the sum of its related item cores by the total number of struts at the examined domain. We used nonparametric tests to compare data. A stepwise multiple regression analysis was conducted to identify the independent variables associated with the QOL score. Cronbach's alpha was used to evaluate internal consistency of questionnaires and an internal consistency estimate of a magnitude of 0.70 was sought. Statistical analyses were performed using SPSS v.18 (SPSS, Chicago, Illinois, USA) and a P value less than 0.05 was considered significant.

## 4. Results

Among the 483 identified patients treated in the period from 2001 to 2009, 124 were eligible and of them 30 deaths were identified and 29 were inaccessible. Therefore, the population available for study consisted of 65 women. The mean duration of phone interviews was 27.5 ± 8.0 minutes. Patients’ demographic and clinical data are presented in Table 1. The average age of participants at study entry was 57.8 ± 11.02 (range 37 to 80) years, and mean post-treatment period was 2.81 ± 1.44; range 1 to 7years. The average score of QOL was 46.9 ± 7.6 percent. The global QOL score was categorized as poor score (0-33), moderate score (34-66), and good score (67-100) which was found in 3 (4.6%), 61 (93.8%), and 1 (1.5%) patients, respectively. The EORTC QLQ-C30 and QLQ-CX24 scores as well as some other assessments among participants are shown in Table 2. Also, the association between QOL score and sub-domains of the questionnaires are indicated in the table. Of the QLQ-C30 sub-domains, QOL score was significantly associated with social functioning in functional scales (P = 0.005), and presence of nausea and vomiting (P < 0.001), short breathing (P = 0.036), lack of appetite (P = 0.021), and sleep disorders (P = 0.006) as well as financial problems (P < 0.001) in symptom scales. Also, QOL score was significantly associated with menopausal symptoms (in QLQ-CX24 questionnaire) (P < 0.001) and peripheral neuropathy (P = 0.005). There were significant associations between the QOL score and the city of birth (P = 0.044) and duration after the treatment (P = 0.017).

**Table 1.** Demographic and Clinical Data (n = 65)

Variable	
**Age, y, Mean ± SD**	
37-80	57.8 ± 11.02
**Education, No. (%)**	
Illiterate	23 (35.4)
Primary school	22 (33.8)
Guidance school	12 (18.5)
High school	6 (9.2)
University	2 (3.1)
**Job, No. (%)**	
Homemaker	62 (95.4)
Employed	3 (4.6)
**Marital status, No. (%)**	
Married	48 (73.8)
Widowed	15 (23.1)
Divorced	2 (3.1)
**Stage of cervical cancer, No. (%)**	
Stage I	32 (49.2)
Stage II	24 (36.9)
Stage III	9 (13.8)
**Age at diagnosis ,y, Mean ± SD**	54.8 ± 11.3
**Duration of treatment, month, Mean ± SD**	3.4 ± 7.0
**Number of treatment sessions, Mean ± SD**	28.9 ± 8.1
**Treatment modality, No. (%)**	
Surgery + Radiotherapy	32 (49.2)
Radiotherapy only	19 (29.2)
Radiotherapy + Chemotherapy	13 (20)
Chemotherapy only	1 (1.5)

**Table 2.** Correlation Between Sub-Domains of the Questionnaires and Global Quality of Life Score

Sub-domains		Range	P value	R
**EORTC QLQ-C30**				
**Functional Scales, Mean ± SD**				
Physical functioning	72.1 ± 26.1	26.7-100	0.142	0.562
Role functioning	75.7 ± 27.1	11.1-100	0.056	0.543
Emotional functioning	55.8 ± 32.6	0-100	0.063	0.737
Cognitive functioning	69.2 ± 21.1	0-33.3	0.109	0.540
Social functioning	91.4 ± 15.2	50-100	0.005	0.272
**Symptom Scales, Mean ± SD**				
Energy/Fatigue	19.6 ± 28.2	0-100	0.230	-0.599
Nausea and vomiting	2.3 ± 10.6	0-50	< 0.001	-0.291
Pain	26.1 ± 34.8	0-66.7	0.708	-0.671
Short of breath	15.4 ± 46.8	0-33	0.036	-0.356
Sleep disturbance	27.2 ± 51.3	0-33	0.006	0.390
Lack of appetite	11.8 ± 30.3	0-100	0.021	-0.362
Constipation	19.5 ± 35.8	0-100	0.454	-0.567
Diarrhea	7.7 ± 42.3	0-33	0.342	-0.061
Financial problems	31.3 ± 37.2	0-100	< 0.001	-0.331
**EORTC QLQ-CX24**				
Symptom experience	13.4 ± 15.4	0-66.7	0.175	-0.191
Body image	36.4 ± 28.4	0-100	0.303	-0.891
Sexual/vaginal functioning (n=16)	70.3 ± 27.2	0-33.3	0.076	0.272
Lymphedema	11.3 ± 4.5	0-100	0.459	0.93
Peripheral neuropathy	23.6 ± 31.0	0-100	0.005	-0.271
Menopausal symptoms	34.9 ± 31.0	0-100	0.001	-0.299
Sexual worry (n=43)	84.9 ± 28.5	0-100	0.113	-0.084
Sexual activity	6.1 ± 15.5	0-66.7	0.438	0.073
Sexual enjoyment (n=16)	39.6 ± 27.8	0-66.7	0.508	0.062
Social support	34.1 ± 20.9	5-87.5	0.687	0.051
Psychological adjustment	53.8 ± 12.0	30-80	0.767	0.038
Spiritual factors	95.8 ± 10.2	30-100	0.954	0.007

## 5. Discussion

Overall, about all of the cervical cancer survivors had reported a good QOL. Similarly, Bradley et al. indicated that QOL in cervical and endometrial cancer patients more than 5 years post-diagnosis was similar to healthy women with no experience of cancer (7) Wenzel et al. showed that overall QOL of cervical cancer survivors was good (8) and it was similar to the general population in Korfage’s study (9). The QOL had a significant direct correlation with the city of birth and the duration after treatment. At the 11-years post-treatment in Bradley’s study, there was no difference between patients and controls (7). The long time post-treatment period could affect the individual adjustment to the condition. Our results indicated that the physical functioning of cervical cancer patients had no impact on the QOL. However, a negative association was observed between QOL score and some symptoms e.g. menopausal symptoms. The presence of menopausal symptoms had a negative impact on QOL in Wenzel’s study (10). Also, they indicated that patients with sexual problems also had a lower QOL. Our study had a few respondents about sexual disorders, so in this aspect, it could not be comparable with other studies. Bradley et al. found no difference between their cancer patients and healthy controls with regard to physical functioning (7). Also, Korfage indicated that problems with sexual activity and other symptoms were observed more among patients who underwent radiotherapy (9). These variations may be due to differences between mean post-treatment period at interview which was less in our population and it may influence on presence of symptoms in the early months after treatment. Also, it can vary from the point of view of the studies about physical functioning. In fact, it reflects the performance status and the capacity of the patient to carry out basic day-to-day activities including motivation, effort, stamina and coordination. It may be due to a special focus on one dimension while neglecting another for generation with some differences between studies. Our study measured the mental health in emotional, cognitive, and self-image states. Mental health was defined as a state of well-being in which every patient realized her own potential, could cope with the normal stresses of life, could work productively, and was able to make a contribution to her community (10). There was no significant correlation between QOL and emotional functioning among our patients. However, some other studies indicated a significant relationship between body image and QOL, (9, 11) as well as a worse score among cancer patients in regard to mental health in comparison with general population (9). Although, some studies similarly found no difference between cancer patients and control group about the effect of emotional functioning on QOL, (7) some others demonstrated that emotional functioning could influence the patients during and after the treatment (12). Korfage et al. showed a direct correlation between the QOL and body image domain and no relationship with cognitive function (9). However, the difference in the age of patients at interview, post-diagnosis period, and cultural variation could result these dissimilarities. Our results demonstrated that there was a relation between the QOL and social functioning and a direct correlation between the QOL and economic conditions. Wenzel stated that patients with improved social support had a higher QOL score (8). However, Bradley et al. and Wenzel et al. indicated no difference between cancer patients and general population in social functioning QOL subscales (7, 8). In conclusion, it is suggested that the scales with lower scores should be the subjects of more precise attention and more efficient and effective intervention in order to provide a better QOL during and after the treatments. Although, the QOL in cervical cancer survivors was good, treatment of related symptoms can influence the QOL and improve the care of cervical cancer survivors.
